# A novel twin‐column continuous chromatography approach for separation and enrichment of monoclonal antibody charge variants

**DOI:** 10.1002/elsc.202000094

**Published:** 2021-05-12

**Authors:** Shu‐Ying Jing, Ce Shi, Hui Yi Leong, Jun‐Jie Yuan, Dong Gao, Hai‐Bin Wang, Shan‐Jing Yao, Dong‐Qiang Lin

**Affiliations:** ^1^ Key Laboratory of Biomass Chemical Engineering of Ministry of Education College of Chemical and Biological Engineering Zhejiang University Hangzhou P. R. China; ^2^ BioRay Pharmaceutical Co., Ltd. Taizhou P. R. China

**Keywords:** charge variant, continuous chromatography, enrichment, monoclonal antibody, separation

## Abstract

Downstream processing of mAb charge variants is difficult owing to their similar molecular structures and surface charge properties. This study aimed to apply a novel twin‐column continuous chromatography (called N‐rich mode) to separate and enrich acidic variants of an IgG1 mAb. Besides, a comparison study with traditional scaled‐up batch‐mode cation exchange (CEX) chromatography was conducted. For the N‐rich process, two 3.93 mL columns were used, and the buffer system, flow rate and elution gradient slope were optimized. The results showed that 1.33 mg acidic variants with nearly 100% purity could be attained after a 22‐cycle accumulation. The yield was 86.21% with the productivity of 7.82 mg/L/h. On the other hand, for the batch CEX process, 4.15 mL column was first used to optimize the separation conditions, and then a scaled‐up column of 88.20 mL was used to separate 1.19 mg acidic variants with the purity of nearly 100%. The yield was 59.18% with the productivity of 7.78 mg/L/h. By comparing between the N‐rich and scaled‐up CEX processes, the results indicated that the N‐rich method displays a remarkable advantage on the product yield, i.e. 1.46‐fold increment without the loss of productivity and purity. Generally, twin‐column N‐rich continuous chromatography displays a high potential to enrich minor compounds with a higher yield, more flexibility and lower resin cost.

**Abbreviations**
AV: acidic variant
BV: basic variant
CEX: cation exchange chromatography
CPB: recombinant carboxypeptidase B
CV: column volume
MES: 2‐morpholinoethanesulfonic acid monohydrate
MP: main peak

## 1 INTRODUCTION

MAbs are one of the most crucial biopharmaceutical drugs which have a great market value [1]. However, various mAb heterogeneities could be formed during the production process, including enzyme catalysis, degradation and modification [2, 3]. Charge variants are the common species of mAb heterogeneities which change on the charges due to Met/Trp oxidation, C‐terminal Lys/Arg, N‐terminal pyroglutamic acid and N‐glycosylation [4, 5, 6]. Compared to the main mAb molecule, charge variants can be divided into two groups, i.e. acidic variants with a lower pI and basic variants with a higher pI [7]. As the result of the differences on number of charge residues and charge distribution on protein surface, many mAb charge variants are reported to have a decreased efficacy and/or lead to unintended side‐effects [8, 9, 10, 11]. Hence, it is necessary to separate and characterize the mAb charge variants for future drug development.

Cation exchange (CEX) chromatography is regarded as the gold standard to separate mAb charge variants [12, 13, 14]. However, the drawbacks like the existence of peak overlap for main mAb and charge variants because of the similar molecular structures and surface charge properties have limited its application. Therefore, many researchers are trying to improve the separation efficiency. Generally, the resin particle size plays the most critical role on the resolution. Besides, the elution gradient and flow rate are also important [15]. Lee et al. [16] compared three resins with different particle diameters (Capto S with 90 μm, CM Sepharose with 90 μm and SP ImpRes with 36–44 μm) for the separation of charge variants of antibody fragments. The results indicated that only SP ImpRes with 36–44 μm diameter was able to achieve 100% pure antibody fragments. Wang et al. [17] reported a two‐step procedure to separate the charge variants of bevacizumab biosimilar. The SP Sepharose High Performance resin with 34 μm diameter was first applied in separating the acidic variants (97.6% purity) and this was followed by the use of Mono S rein with 10 μm diameter. The main mAb with a purity of 94.6% and basic variants with a purity of 88.7% were attained. Zhang et al. [18] investigated the isolation and enrichment of mAb charge variants using CEX displacement chromatography. The recovery of acidic variants, main mAb and basic variants in 90% purity were 69%, 84%, and 78%, respectively. Jing et al. [19] used Nano SP‐15L resin with 15 μm diameter to separate charge variants and pure main mAb with a yield of 56.5% was obtained. The yield was low but the main mAb can be obtained in high purity. Nevertheless, 100% purity of mAb charge variants are yet to be hardly achieved due to their similar structure and relative low content in the feedstock. Due to low recovery, repetition on the separation procedure in numerous times is required to have sufficient charge variants for further detection and characterization. This is laborious, time consuming and required large amount of mAb feedstock.

**PRACTICAL APPLICATION**

Continuous chromatography is the trend in downstream mAb production which shows a higher productivity and flexibility as well as cost efficiency. This study focused on the application of a novel twin‐column continuous chromatography (called N‐rich mode) to separate and enrich mAb charge variants. Compared with the scaled‐up batch‐mode cation exchange chromatography under the same productivity, the N‐rich process showed a higher yield with much smaller column and lower cost needed. In addition, continuous chromatography was proved to have a better enrichment capability on minor components.

In recent years, continuous chromatography is a promising trend in the biopharmaceutical industry, which has the potential in enhancing the process efficiency and productivity [20, 21, 22, 23, 24]. Several novel modes were proposed for the continuous polishing. Based on the principle of recycling the overlapped region between main peak and the weakly/strongly‐binding components, Steinebach et al. [25, 26, 27] proposed the multicolumn counter‐current solvent gradient purification (MCSGP) to improve the recovery of main protein with high purity. Persson et al. [28] reported a two‐column batch‐to‐batch recirculation process and the results showed that the yield was increased 3.4 times compared to the batch run after 20 cycles. Khanal et al. [29] suggested the multi‐column displacement chromatography to separate mAb charge variants by using the basic variants and main protein as the displacers. The main protein was enriched from 65% to 90% with a yield of above 90%. All in all, continuous chromatography is demonstrated to be a useful tool to improve the tradeoff of purity and yield of main protein in the mixture.

Other than that, continuous chromatography can also be utilized for separation and enrichment of minor components. Thomas et al. [30] proposed a novel twin‐column continuous chromatography, called N‐rich mode, to enrich target components. For the N‐rich process, the region of target peak is recycled to the next column to accumulate the target compounds. N‐rich process is regarded as a crucial approach for the enrichment of minor compounds with much higher yield under the desired purity demand, which has huge potential to decrease the cost of columns, time and be more automated. In the present work, N‐rich process would be tested to separate and enrich mAb charge variants. The process design would be studied, and the operation conditions (including buffer system, flow rate and elution gradient slope) would be optimized to ensure both high purity and yield. To clarify the reliability and advantages of N‐rich process, a traditional scaled‐up batch‐mode CEX would be tested and used as the control. A comparison on the N‐rich and batch process would be provided, particularly in the aspect of yield, productivity, column size and process time.

## 2 MATERIALS AND METHODS

### 2.1 Materials

Four prepacked columns with Proteomix SCX‐NP10 resin were provided by Sepax Technologies, Inc. (Suzhou, China) with different sizes (i.e. 4.6 mm diameter and 250 mm length, 10 mm diameter and 250 mm length, 21.2 mm diameter and 250 mm length, 10 mm diameter and 50 mm length). Human IgG1 mAb sample with 8.71% acidic variants and 47.49% basic variants was kindly provided by a local biopharmaceutical company. The mAb mass weight is around 148 kDa and the pI is about 8.7. The CPB treatment was used to remove the C‐terminal Lys [7, 31‐32]. 15 μL CPB were added into 1 mL mAb sample with 1 g/L and kept for 30 min under 37℃. The proportion of main mAb and acidic variants was 88.47% and 11.53%, respectively after CPB treatment.

Antibodix WCX‐NP5 analytical column (4.6 mm diameter × 250 mm length) was purchased from Sepax Technologies, Inc. (Suzhou, China). Sodium chloride, sodium dihydrogen phosphate dehydrate and disodium hydrogen phosphate dodecahydrate were purchased from Sinopharm Chemical Reagent Co., Ltd (Shanghai, China). 2‐Morpholinoethanesulfonic acid monohydrate (MES) was acquired from Sigma‐Aldrich. Recombinant carboxypeptidase B (CPB) was obtained from Shanghai Yaxin Biotechnology Company (Shanghai, China). All other chemicals used in this study were of analytical grade.

### 2.2 Charge variants separation with batch chromatography

The batch CEX chromatography separation process was carried out using an AKTApurifier system (GE Healthcare, Uppsala, Sweden). Three Proteomix SCX‐NP10 prepacked columns with 250 mm length and different diameters were tested with the column volumes (CV) of 4.15, 19.63, and 88.20 mL, respectively. The experimental details are listed in Table 1. For all experiments, the column was equilibrated with 2 CV equilibration buffer, then different amount of protein samples were loaded for the process optimization. After 2 CV strip with the equilibration buffer, the column was eluted with a gradient of 0‐100% elution buffer in 12 CV. Last, the column was regenerated with 2 CV 1.0 M NaCl and re‐equilibrated with 2 CV equilibration buffer.

**TABLE 1:** Experimental conditions of mAb charge variants separation with batch and N‐rich chromatography process

	Equilibration buffer	Elution buffer	Loading (mg/mL resin)	Gradient slope (mM/CV)	Flow rate (cm/h)	Column size (mm)	Column volume (mL)
Batch	20 mM PBS, pH 6.5 0.05 M NaCl	20 mM PBS, pH 6.5 0.09 M NaCl	0.1‐0.4	3.33	230	4.6 × 250	4.15
						10 × 250	19.63
						21.2 × 250	88.20
N‐rich	20 mM MES, pH 5.6 0.1 M NaCl	20 mM MES, pH 5.6 0.25 M NaCl	0.1	6.25	230	10 × 50	3.93

### 2.3 Charge variants enrichment with continuous chromatography

The N‐rich mode continuous chromatography enrichment process was carried out with Contichrom CUBE combined 100 system (ChromaCon AG, Zurich, Switzerland). Two prepacked columns (10 mm diameter and 50 mm length) with Proteomix SCX‐NP10 resin were used. The experimental conditions are listed in Table 1.

Batch CEX with one column was first used to evaluate the appropriate separation conditions. MES buffer was used. The column was equilibrated with 3 CV equilibration buffer and the protein sample was loaded with 0.1 mg/mL resin. Then the column was washed with 3 CV equilibration buffer and eluted with a gradient of 0‐50% elution buffer in 12 CV. Last, the column was regenerated with 3 CV elution buffer and re‐equilibrated with 3 CV equilibration buffer. The optimum performance of the batch CEX was used as a design base for the N‐rich process.

**FIGURE 1:**
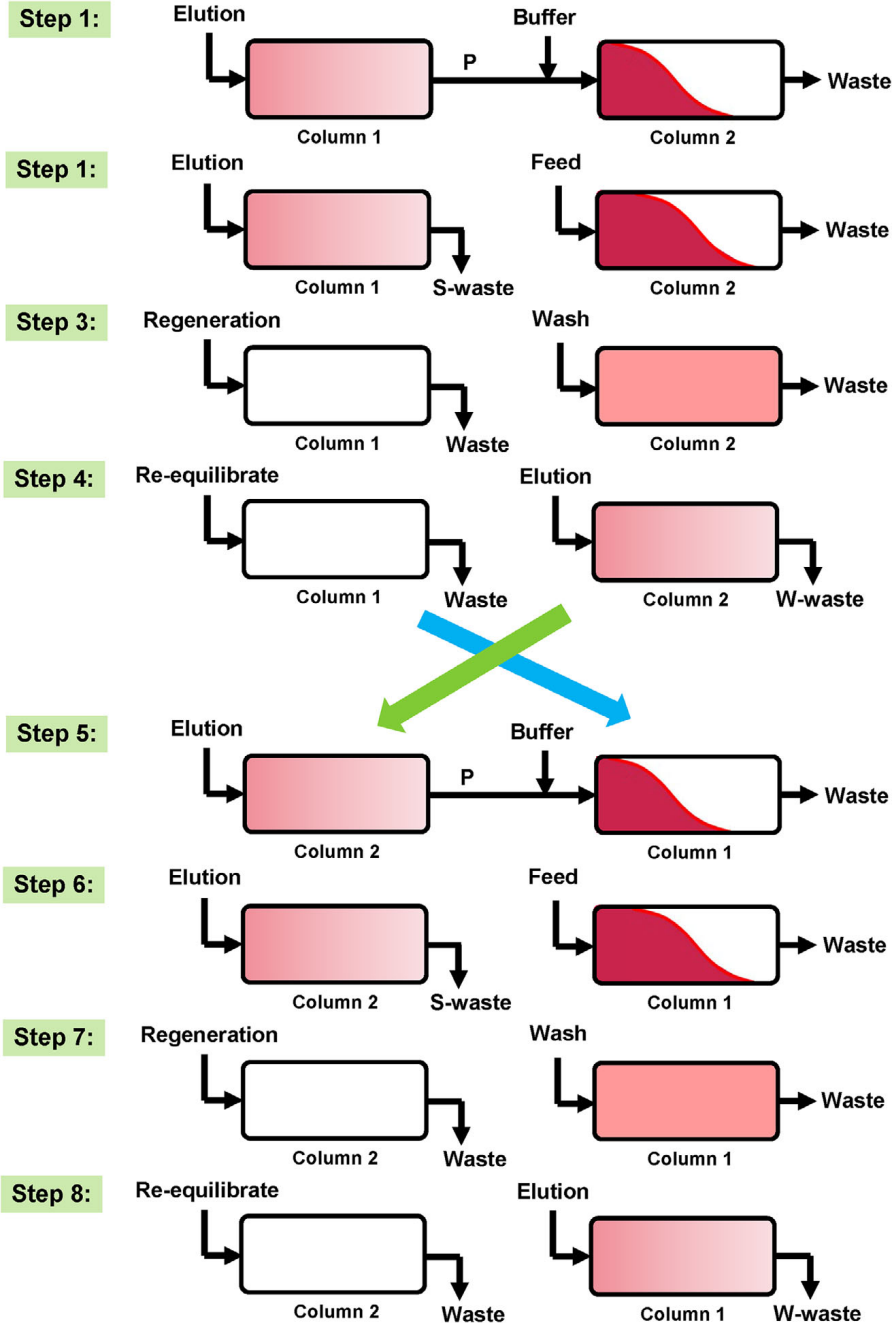
Scheme of accumulation phase of N‐rich process with twin‐column continuous chromatography

An entire N‐rich process consists of three phases, i.e. accumulation phase, separation phase and elution phase. The recycling region was accumulated by reloading to the next column internally together with fresh feedstock in the first accumulation phase. The scheme of accumulation phase is illustrated in Figure 1. In the first step, two columns are interconnected in series and the target component peak eluted from the 1# column is pumped into the 2# column. Meanwhile, the elution flow is diluted by the equilibration buffer to ensure the target component can be adsorbed in the 2# column. Then, the two columns are disconnected. The 1# column undergoes the elution and regeneration steps for strongly‐binding components and the 2# column is loaded with fresh feedstock as shown in Steps 2 and 3 in Figure 1. After the loading and wash step, the 2# column enters the gradient elution step to elute the weakly‐binding components while the 1# column undergoes the re‐equilibration step (Step 4 in Figure 1). When the target component is eluted out from the 2# column, the 1# column is connected to the 2# column to capture the target component with the on‐line dilution, as shown in Step 5 (Figure 1). Although Step 5 is noted to be almost similar to Step 1, the two columns change the turn. The procedure will continue and finish a cycle after Steps 6, 7, and 8. After several cycles are being performed, the target component can be enriched and separated in a relatively easy way, especially for small amount of component in a mixture. In the separation phase, the recycling region was reloaded to the next column without the supplement of fresh feedstock. Thus, the undesired compounds were removed without the loss of the target compounds. In the elution phase, the target compounds were eluted, and the gradient slope and flow rate were optimized for an effective separation performance.

### 2.4 Analysis

The Antibodix WCX‐NP5 analytical column and CXTH‐3000U HPLC system (Beijing Tong Heng Innovation Technology Co., Ltd., Beijing, China) were used for the sample analysis. The flow rate was set at 0.8 mL/min. The equilibration buffer and elution buffer were 0.05 M NaCl in 20 mM phosphate buffer (pH 6.5) and 0.1 M NaCl in 20 mM phosphate buffer (pH 6.5), respectively. The gradient from 0% to 100% elution buffer in 30 min was used. The protein response was detected using UV detector at 280 nm.

## 3 RESULTS AND DISCUSSION

### 3.1 Charge variants separation with traditional batch‐mode chromatography

Batch CEX chromatography is the traditional and conventional approach to separate mAb charge variants. A 4.15 mL Proteomix SCX‐NP10 prepacked column was tested first, and 20 mM PBS buffer (pH 6.5), 230 cm/h and 3.33 mM/CV slope were chosen as the elution conditions after the process optimization. To balance the separation resolution and process productivity, the load amount was optimized with 4.15 mL column, and then the process was scaled up to a larger column. First, the mAb sample without CPB treatment was used, and the proportion of main mAb, acidic variants and basic variants were 43.80%, 8.71% and 47.49%, respectively.

**FIGURE 2:**
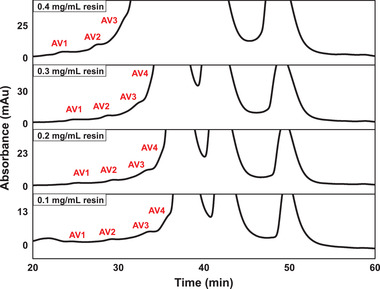
Chromatograms of the charge variants separation with 4.15 mL Proteomix SCX‐NP10 prepacked column under different load amounts. AV, acidic variant

The chromatograms of different load amounts (0.1‐0.4 mg protein/mL resin) with 4.15 mL column are shown in Figure 2. Only three peaks of acidic variants were observed under the load amount of 0.4 mg protein/mL resin. Four acidic variants could be separated with the load amount of 0.3 and 0.2 mg protein/mL resin, and the resolution was obviously better with the load amount of 0.2 mg protein/mL resin. When the load amount was further decreased to 0.1 mg protein/mL resin, the improvement on the resolution was limited. By considering both the resolution and productivity, the load amount of 0.2 mg protein/mL resin (corresponding 0.83 mg protein for 4.15 mL column) was chosen as the best condition for scaling up study.

**FIGURE 3:**
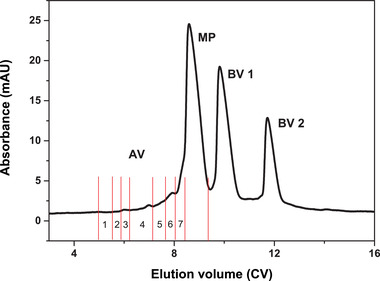
Chromatogram of the charge variants separation with 88.20 mL column under the load amount of 0.2 mg protein/mL resin. AV, acidic variant; BV, basic variant; MP, main peak

**FIGURE 4:**
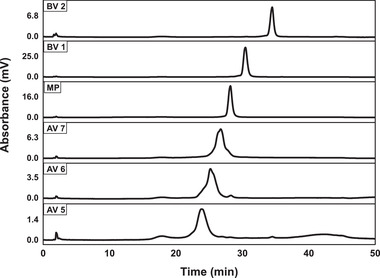
HPLC analysis of the fractions collected during the separation with 88.20 mL column under the load amount of 0.2 mg protein/mL resin. AV, acidic variant; BV, basic variant; MP, main peak

The process was scaled up to 19.63 and 88.20 mL columns with the load amount of 0.2 mg protein/mL resin. Both the processes were noted to show a consistent separation performance with that of the above test with 4.15 mL column. For the separation with 88.20 mL column, 17.64 mg protein was loaded in one batch, which was 21.25 times the amount of 4.15 mL column. The elution fractions are shown in Figure 3. Seven individual acidic variants (AV1‐AV7) were separated. The elution fractions were collected and analyzed by HPLC. The contents of AV1‐AV4 were all less than 0.1 mg, and the contents of AV5‐AV7 were 0.16, 0.23, and 0.45 mg, respectively. The HPLC result of three acidic variants (AV5‐AV7), main protein and two basic variants (BV1‐BV2) are shown in Figure 4. The results indicated that the scaled‐up batch process could separate individual acidic variants with near 100% purity. However, the yield was only 69.07%.

In addition, the CPB treated mAb sample was investigated further for the separation of acidic variants with 88.20 mL column. The proportion of acidic variants increased to 11.53% after CPB treatment, but the yield of acidic variants was as low as 59.18% under the same separation conditions.

### 3.2 Charge variants enrichment with twin‐column continuous chromatography

To transfer the normal batch mode to the N‐rich continuous chromatography, buffer system, column length and elution gradient should be re‐optimized first to match the requirements of continuous process. Then the N‐rich process was carried out to enrich and separate acidic variants after the design of enrichment strategy. The CPB treated sample was used for the N‐rich process.

#### 3.2.1 Buffer system

First, the same PBS buffers (pH 6.5) were used as that in batch chromatography. However, the N‐rich process could not perform well. This is most probably due to the salt concentration difference between the beginning and end gradient for the target acidic variants was about 12 mM, corresponding to the conductivity difference of about 0.8 mS/cm. The limited conductivity range would cause high difficulty on the process control for N‐rich continuous chromatography. After the optimization, the PBS buffer (pH 6.5) was changed to the MES buffer (pH 5.6), the salt concentration difference increased to about 20 mM, corresponding to the conductivity difference of 1.2 mS/cm, which caused a good enrichment performance of the acidic variants.

#### 3.2.2 Column length and elution gradient

As shown in Figure 1, two columns should be interconnected for the recycling of target component for N‐rich process. The column length is limited due to the limitation of system pressure. Thus, the column with 10 mm diameter and 50 mm length was used to reduce the pressure in the interconnected step of continuous chromatography. A shorter column will certainly weaken the separation performance, so the separation conditions need to be optimized to improve the separation performance, including increasing the residence time and decreasing the load amount. The optimized separation conditions are listed in Table 1. It could be found that the elution gradient was increased to 6.25 mM/CV to avoid the long process time for N‐rich process. The load amount was decreased from 0.2 to 0.1 mg/mL resin to ensure the acceptable separation performance.

#### 3.2.3 Enrichment strategy

**FIGURE 5:**
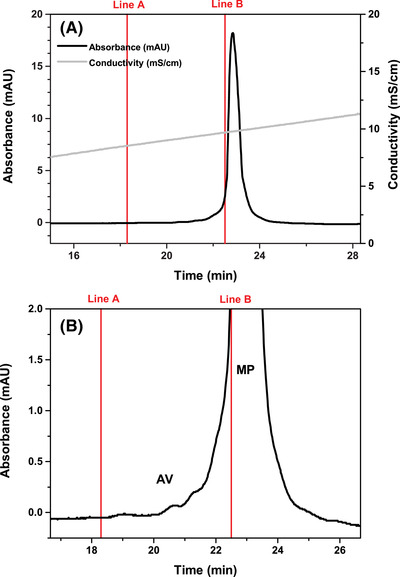
Design chromatogram for N‐rich process. (A) Full chromatogram; (B) Particular region to specify on recycling phase. AV, acidic variant; MP, main peak

Although the separation performance had improved after the process optimization but the overlapping between acidic variants and main mAb was still a main issue. The elution profile is showed in Figure 5. It is relatively difficult to accumulate the individual acidic variants due to low content of acidic variants and the overlapping between acidic variants and main mAb. So the enrichment strategy was set to have the recycling region as large as possible in the accumulation phase which could reduce the loss of acidic variants. Then, the acidic variants could be separated and obtained individually in the last elution phase of N‐rich process. As shown in Figure 5, Line A and Line B represent two time points and the region between Line A and Line B was regarded as the recycling region. Specifically, the choosing of Line A need to ensure that there was no acidic variants eluted earlier before Line A and the choice of Line B needs to ensure that all acidic variants eluted before Line B. Thus, 18.3 min was chosen as Line A and 22.5 min was chosen as Line B (as shown Figure 5). In the last elution phase of N‐rich process, a slower flow rate of about 115 cm/h and a shallower elution gradient of about 2.08 mM/CV were used to provide a better separation of the accumulated acidic variants and main protein, and thereby realize pure acidic variants.

#### 3.2.4 N‐rich process for charge variants enrichment

**FIGURE 6:**
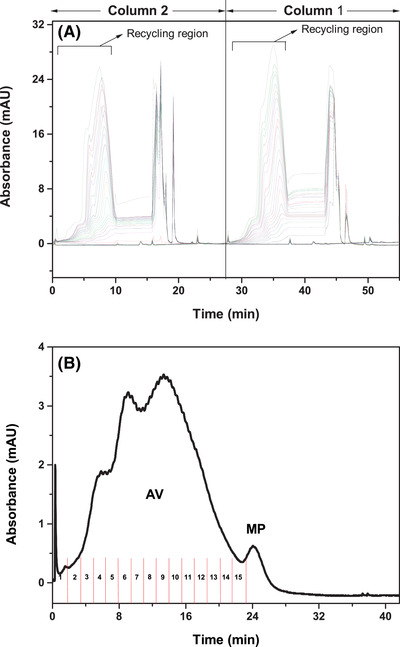
Chromatograms of 22‐cycle N‐rich process. (A) Accumulation phase; (B) Elution phase. AV, acidic variant; MP, main peak

**FIGURE 7:**
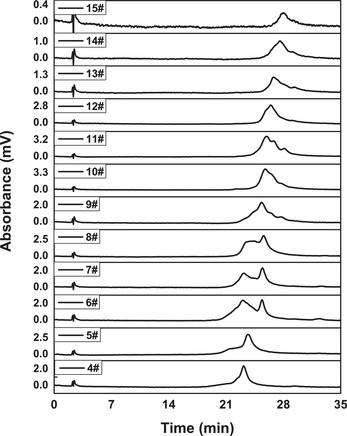
HPLC analysis of the acidic variant fractions collected in N‐rich process

Based on the optimized conditions mentioned above, a 3‐cycle N‐rich process was first performed to ensure the continuous chromatography could work well. Subsequently, the cycle number increased gradually to 22 cycles. The load amount was similar to that of the batch‐mode separation with 88.20 mL column. The chromatograms of 22 cycles of N‐rich process are shown in Figure 6(A). For each cycle, the recycling region increased obviously with the increase of cycle numbers, which demonstrated a desired accumulation of the acidic variants. The elution profile of the accumulated recycling region is provided in Figure 6(B). It could be observed that the main mAb peak only had a rather low proportion as compared to the accumulated acidic variants peaks. The elution factions were collected and analyzed by HPLC. In Figure 7, there was mostly one type of acidic variants for NO. 4 ‐ NO. 5 which was eluted at about 23.4 min. For NO. 6 ‐ NO. 8, two types of acidic variants were eluted together which had the certain range of overlapping. And there was one type of acidic variants mainly for NO. 9 ‐ NO. 15. Based on the HPLC analysis, a total of 1.33 mg acidic variants were obtained after 22‐cycle enrichment, which showed a recovery of 86.21%.

### 3.3 Comparison between batch‐mode and N‐rich process for charge variants enrichment

A comparison between batch‐mode chromatography and N‐rich continuous process was discussed for charge variants separation and enrichment in term of column size, process time and yield. The results with the same CPB treated sample are listed in Table 2.

**TABLE 2:** Comparison between acidic variants enrichment with the use of batch and N‐rich continuous chromatography processes

	Column volume (mL)	Load amount (mg/mL resin)	Total load amount (mg)	AV obtained (mg)	Yield (%)	Productivity (mg/L/h)	Process time (min)	Cycle number
Batch	88.20	0.20	17.64	1.19	59.18	7.78	104	–
N‐rich	7.86 (3.93*2)	0.10	17.54	1.33	86.21	7.82	1299	22
Batch‐1	7.86	0.10	0.79	0.05	59.18	3.67	104	–
Batch‐2	7.86	0.10	17.38	1.17	59.18	3.90	2288	22

#### 3.3.1 Column size

In Batch and N‐rich process, the column volume was 88.20 and 7.86 mL, respectively, which meant that it was 11.22 times larger in Batch compared to N‐rich process. Specifically, a 22‐cycle N‐rich process had the total load amount of about 17.5 mg as the Batch process. Thus, N‐rich continuous process could be carried out using much smaller columns which meant more advantages on column size, process cost and flexibility without the loss of separation performance.

#### 3.3.2 Process time

The process time for Batch mode was only 104 min, i.e. 12.49 times shorter than that of N‐rich process. However, time‐saving along with higher expense by using much larger column size is not favorable. The situation can be evaluated by using smaller columns. Batch‐1 represents the batch CEX process with the column volume of 7.86 mL, the load amount of 0.1 mg/mL resin and the yield of about 59.18%. Batch‐1 needs to be repeated 22 times, which is represented as Batch‐2, to achieve the same total load amount about 17.5 mg with N‐rich process. Thus, the process time of Batch‐2 would increase to 2288 min, i.e. 1.76 times longer than N‐rich process. Besides, unlike N‐rich, batch process has to concentrate the collective proteins, which not only cost a tremendous amount of time and effort but also cause inevitable protein loss in the repeated ultrafiltration concentration step. In short, although the scaled‐up batch can obtain a relatively large amount of target protein in a shorter time, the automated N‐rich process represents a more efficient way on time and labor in performing the tedious separation and analysis with common small columns.

#### 3.3.3 Yield

The content of acidic variants attained was about 1.33 mg for N‐rich process, i.e. 1.12 times higher than Batch process. The yield of acidic variants was 86.21% for N‐rich process, in which showed an increment of 1.46 times compared to that of the Batch process. The productivities of N‐rich and Batch processes were comparable at about 7.80 (mg/L/h) even though the loading amount of N‐rich process was only a half of Batch process. Zhang et al. [18] used cation exchange displacement chromatography for enrichment and separation of mAb charge variants. The yield of acidic variants was about 69% using 10 μm Mono S columns with Expell SP1 displacer, which is near the value of Batch process in the present work. Hence, N‐rich process displays obvious merits in acidic variants enrichment by providing a promising recovery yield with high productivity and purity.

Although many researchers have successfully separated mAb charge variants, the goal of these papers mainly focused on the purification of main mAb, i.e. reduce the content of charge variants in mAb sample through the optimization of separation conditions [16, 19, 25, 26, 27, 28, 29]. For example, Jing et al. [19] separated charge variants to obtain 100% pure main mAb with the optimized separation conditions. Although several acidic variants could be separated in elution profile, the acidic variants were not obtained with desired purity and yield. Besides, many researchers were concerned about the improvements of mass technology for the characterization of mAb charge variants rather than the preparative separation of mAb charge variants [33, 34, 35, 36]. Markus et al. [33] reported mAb charge variants separation using a Source 15S cation exchange column, but the yield and productivity were not mentioned, because the main objective was to develop an online cation‐exchange chromatography native electrospray mass spectrometry method (CEC‐UV‐MS) for charge variant monitoring. Therefore, it is still a tough task to enrich and separate mAb charge variants with high purity and yield. The results in the present work demonstrated that N‐rich continuous process as a new method could provide a good choice for the enrichment of minor components in the mixture such as mAbs charge variants, which could improve the yield obviously without the loss of productivity and purity.

## 4 CONCLUDING REMARKS

The separation and enrichment of mAb charge variants using a novel twin‐column continuous chromatography (N‐rich process) were studied. After the process optimization, a total amount of 17.54 mg protein was loaded with two 3.93 mL columns, and 1.33 mg acidic variants were obtained with the yield of 86.21% after a 22‐cycle run. A comparison study with the traditional scaled‐up batch‐mode CEX chromatography was performed. For the batch process with 88.20 mL column, 1.19 mg acidic variants were obtained with the recovery of 59.18% under the similar total load amount as the N‐rich process. By comparing batch and continuous processes, N‐rich process showed significant advantages, for which the column size could decrease 11.22 times and the yield could increase 1.46 times. In general, N‐rich process is suitable particularly for the enrichment of minor components in the mixture such as mAbs charge variants, which could improve the yield obviously without the loss of productivity and purity. Moreover, N‐rich process is more flexible, cost effective and can enhance the process automatization.

## CONFLICT OF INTEREST

The authors have declared no conflict of interest.

## Data Availability

The data that support the findings of this study are available from the corresponding author upon reasonable request.
